# A Promising Attenuated Rhabdovirus Vaccine Candidate Conferring Dual-Route Protection Against MSRV Disease in Largemouth Bass (*Micropterus salmoides*)

**DOI:** 10.3390/vaccines13060645

**Published:** 2025-06-16

**Authors:** Xiaozhe Fu, Wenxian Li, Minghui Kong, Hongru Liang, Qiang Lin, Yinjie Niu, Xia Luo, Baofu Ma, Jin Zhou, Ningqiu Li

**Affiliations:** 1 Key Laboratory of Fishery Drug Development, Ministry of Agriculture and Rural Affairs, Guangdong Province Key Laboratory of Aquatic Animal Immune and Sustainable Aquaculture, Pearl River Fishery Research Institute, Chinese Academy of Fishery Sciences, Guangzhou 510380, China; fuxiaozhe@prfri.ac.cn (X.F.); lwx1369782725@163.com (W.L.); kongm2022@163.com (M.K.); hrliang@prfri.ac.cn (H.L.); linq@prfri.ac.cn (Q.L.); nyj@prfri.ac.cn (Y.N.); luoxia@prfri.ac.cn (X.L.); mabf@prfri.ac.cn (B.M.); 2 Shenzhen International Graduate School, Tsinghua University, Shenzhen 518055, China

**Keywords:** *Micropterus salmoides*, largemouth bass rhabdovirus, attenuated, live vaccine

## Abstract

Background/Objectives: Largemouth bass rhabdovirus (*Micropterus salmoides* rhabdovirus, MSRV) disease causes high mortality in largemouth bass farming. Therefore, vaccine development is critical for largemouth bass prevention against MSRV. Methods: An attenuated strain, denoted as MSRV-0509, was selected through intraperitoneal injection and immersion challenge assays, followed by plaque purification. The biological characteristics of MSRV-0509, including optimal inoculation dose, replication kinetics, thermostability, pH resistance, chloroform tolerance, and storage viability, were determined via viral titration. Spatiotemporal distribution patterns in largemouth bass post-intraperitoneal injection or immersion infection were quantified by qPCR. Immunoprotective efficacy was evaluated through intraperitoneal and immersion vaccination. Mechanistic insights were explored via relative qPCR and serum neutralization assays. Safety was assessed by single-dose overdose immunization and virulence reversion experiments. Results: An attenuated strain MSRV-0509 was screened through a challenge assay, exhibiting complete avirulence in largemouth bass compared to the virulent strain SCRV-T6. MSRV-0509 demonstrated optimal replication at low MOI (0.0001) in CPB cells, with peak titers (10^8.3^ TCID_50_/mL) at 96 h post-infection. The virus showed susceptibility to high temperatures, lipid solvents and acidic conditions, with prolonged stable storage viability at −80 °C. Tissue distribution revealed the spleen as the primary target after intraperitoneal injection, while immersion restricted infection to gills, with rapid clearance by 3–6 dpi. Vaccination trials identified 5 × 10^2^ TCID_50_/fish via intraperitoneal injection and 10^6.0^ TCID_50_/mL via immersion as effective immunizing doses, providing 100% relative survival post-challenge. Immune gene expression and serum neutralization showed Th1 and Th2 activation via intraperitoneal injection (elevated IL-12, IFN-γ, IL-10, IgM), whereas only the Th1 response was activated after vaccine immersion. No abnormality and mortality were observed in single overdose vaccination and virulence reversion experiments, confirming that MSRV-0509 was safe. Conclusions: These results proved that MSRV-0509 could be a promising vaccine candidate to protect largemouth bass from MSRV disease.

## 1. Introduction

The largemouth bass (*Micropterus salmoides*) is a major freshwater aquaculture species in China, with production reaching 888,030 tons in 2023, accounting for 99% of the global harvest [1]. However, disease outbreaks pose a significant challenge to its farming, particularly viral infections such as MSRV [2], largemouth bass ranavirus (LMBV) [3], and largemouth bass Birnavirus (LBBV) [4]. Among these, MSRV is highly virulent, causing acute mortality with rates reaching 80% in infected fry [5,6]. MSRV belongs to the family *Rhabdoviridae*, subfamily *Alpharhabdovirinae*, and genus *Siniperhavirus* [7]. It is an enveloped, single-stranded negative-sense RNA virus, with a genome encoding five structural proteins: the nucleoprotein (N), phosphoprotein (P), matrix protein (M), glycoprotein (G), and RNA-dependent RNA polymerase (L). Given the lack of effective antiviral treatments, vaccination remains the most promising strategy for disease control in aquaculture.

Vaccination serves as a highly effective strategy for controlling viral diseases in aquaculture [8,9]. The most prevalent delivery methods include injection and immersion, while oral administration remains less commonly utilized globally [10]. For small fry, immersion vaccination is required, while larger fingerlings or broodstock need injection vaccination. Live-attenuated vaccines have demonstrated superior efficacy compared to inactivated or subunit vaccines due to their ability to mimic natural viral infections, be delivered via immersion or injection, and induce strong and long-lasting immune responses, including both cellular and humoral immunity [11]. So far, several live-attenuated vaccines have been successfully approved for aquaculture applications, including the avirulent Grass Carp Reovirus (GCRV) vaccine in China, live *Edwardsiella tarda* and genetically engineered *Vibrio anguillarum* vaccines for turbot (*Scophthalmus maximus*) in China, the modified live *Flavobacterium columnare* vaccine for channel catfish (*Ictalurus punctatus*) in the United States, and the attenuated Koi Herpesvirus (KHV) vaccine for common carp (*Cyprinus carpio*) in Israel [10,12,13]. However, a major concern with live vaccines is the potential risk of virulence reversion, which could compromise vaccine safety [5]. Consequently, the generation of an attenuation-stable MSRV strain without virulence reversion potential is crucial for creating a safe live MSRV vaccine for largemouth bass.

In this study, we successfully screened an attenuated MSRV strain (MSRV-0509) and evaluated its biological characteristics, safety, and immunogenicity in largemouth bass. The results supported its potential as a safe and effective live-attenuated vaccine candidate against MSRV infection. This study provides the first evaluation of an attenuated rhabdovirus vaccine for largemouth bass, offering valuable insights into its application in aquaculture disease prevention.

## 2. Materials and Methods

### 2.1. Fish, Cells and Virus

Juvenile largemouth bass (4.34 ± 0.17 cm, 1.81 ± 0.33 g) were acclimated in flow-through tanks with continuous aeration and fed commercially available feed pellets daily. Water temperature was regulated at 28-30°C throughout the experimental period.

The Chinese perch brain (CPB) cell line was established as previously described [14]. CPB cells were cultured in Leibovitz’s L-15 medium (Biosharp, Hefei, China) supplemented with 8% fetal bovine serum (FBS, ExCell, Suzhou, China) at 28 °C under standard culture conditions.

The MSRV strain was originally isolated from an asymptomatic largemouth bass and stored in our laboratory. The SCRV-T6 strain, a highly virulent isolate previously characterized in our laboratory [15], was used as a reference virus. Both viruses were propagated in CPB cells cultured in Leibovitz’s L-15 medium supplemented with 2% FBS. Viral supernatants were harvested, clarified by centrifugation (3000× *g*, 10 min), aliquoted, and stored at −80 °C until further use. Viral titers were determined using the Reed–Muench method.

### 2.2. Screening of the Attenuated Rhabdovirus Vaccine Candidate

#### 2.2.1. The Attenuated Strain Determination

A total of 110 largemouth bass were randomly divided into eleven groups, including ten experimental groups and one control group (10 fish per group per tank containing 50 L of water). MSRV and SCRV-T6 were serially diluted 10-fold (10^1^ to 10^5^) for use in challenge experiments. The five experimental groups were injected intraperitoneally with MSRV at a dose of 5 × 10^5.57^, 5 × 10^4.57^, 5 × 10^3.57^, 5 × 10^2.57^, or 5 × 10^1.57^ TCID_50_/0.05 mL per fish, and the other five experimental groups were injected intraperitoneally with SCRV-T6 at a dose of 5 × 10^6^, 5 × 10^5^, 5 × 10^4^, 5 × 10^3^, 5 × 10^2^ TCID_50_/0.05 mL/fish. Fish in the control group received an intraperitoneal injection of 0.05 mL PBS/fish. All fish were maintained in aquaria at 28 °C with continuous aeration for a 14-day observation period. Mortality was monitored and recorded twice daily, with dead individuals immediately removed to maintain water quality. The mixed tissues of liver, kidney, and spleen from dead fish were used to determine viral load using a quantitative real-time PCR (qPCR) assay.

#### 2.2.2. Purification of Attenuated Rhabdovirus Isolate by Plaque Assay

CPB cells were cultured in 6-well tissue culture plates (Corning, NY, USA) until reaching 90–100% confluency. Monolayers were inoculated with serially diluted viral suspensions (10-fold increments) and incubated for 1 h at 28 °C. After removing the inoculum, cells were overlaid with 2 × DMEM-based agar (Jinuo, Hangzhou, China; phenol red-free) and incubated at 28 °C, 5% CO_2_ until plaque formation. Plaque development was monitored daily by light microscope. When plaques reached 2–3 mm in diameter, 0.01% neutral red solution (MP Biomedicals, Santa Ana, CA, USA) was added for 1–3 h (dark conditions). Following staining, individual plaques were excised and subjected to five sequential rounds of plaque purification.

#### 2.2.3. Virulence Assessment of Clonal Strain by IP Injection and Immersion

Largemouth bass were randomly divided into challenge and control groups (*n* = 30 per group). For IP injection groups, each fish was injected with 0.05 mL of viral suspension at doses of 5 × 10^3^, 5 × 10^2^, or 5 × 10^1^ TCID_50_/fish, while the control group was injected with 0.05 mL PBS (pH7.4). For immersion groups, fish were immersed in 5 L of virus suspension at concentrations of 10^6^, 10^5^, 10^4^, 10^3^, or 10^2^ TCID_50_/mL for 1 h, followed by a 5 min rinse in clean water before being transferred back to their respective aquaria. The control group was exposed only to clean water instead of viral suspension.

### 2.3. Biological Characteristics of the Clonal MSRV-0509 Strain

#### 2.3.1. Optimization of Inoculation Dose

Viral inoculation was performed at different multiplicities of infection (MOIs) of 0.0001, 0.001, 0.01, 0.1, 0.5, and 1.0. When cytopathic effects (CPEs) reached 90%, cells and supernatants were harvested, then subjected to two freeze–thaw cycles, and subsequently, supernatants were used for viral titer determination.

#### 2.3.2. Growth Kinetics Analysis in CPB Cells

The clonal strain MSRV-0509 was used to infect confluent CPB cell monolayers at an MOI of 0.0001. Cell culture supernatants were collected every 12 h post-infection until 120 h. The viral titer at each time point was determined by a TCID_50_ assay, and a growth kinetics curve was plotted based on the titration results.

#### 2.3.3. Physicochemical Characterization

Thermal stability test: Aliquots of MSRV-0509 virus suspension were incubated in a water bath at 34 °C, 40 °C, 46 °C, 52 °C, 56 °C, or 60 °C for 30 min (with a 28 °C control group). Viral titers were then assessed by the TCID_50_ assay.

Chloroform sensitivity test: Virus suspension was treated with 4.8% (*v*/*v*) chloroform (final concentration), vortexed, and incubated at room temperature (25–28 °C) for 10 min. After centrifugation at 3000 rpm for 10 min, the supernatant was collected for TCID_50_ determination.

Acid/alkali resistance test: The pH of MSRV-0509 virus suspension was adjusted to 3.0, 5.0, or 9.0 using 1 M HCl (control: L-15 medium with equivalent volume). Samples were incubated at 25 °C for 2 h, neutralized to pH 7.2 with NaOH, and titrated by TCID_50_ assay.

Virus Storage Stability: Aliquoted viral stocks were stored at 4 °C, −20 °C, and −80 °C, and viral titers were measured every month to assess storage stability.

#### 2.3.4. Tissue Distribution and Kinetics of MSRV-0509 in Largemouth Bass via Intraperitoneal (IP) Injection and Immersion

A total of 80 healthy largemouth bass were randomly divided into two experimental groups (n = 40 per group) as follows: (1) IP injection group: Fish were injected with 50 μL of MSRV-0509 suspension (10^4^ TCID_50_/mL, equivalent to 5 × 10^2^ TCID_50_/fish). (2) Immersion group: Fish were exposed to viral suspension (10^6^ TCID_50_/mL) for 1 h, followed by 5 min rinsing in clean water before returning to rearing tanks. At 1, 2, 3, 4, 5, 6, 7, and 14 days post-immunization (dpi), the brain, heart, liver, spleen, head kidney, intestine, gill, stomach, skeletal muscle, and skin of five fish per group were sampled. A total of 20 mg of each tissue was homogenized for total RNA extraction. MSRV-0509 copy numbers were determined by qPCR in our lab.

### 2.4. Evaluation of Protective Efficacy of Attenuated Vaccine Candidate

#### 2.4.1. IP Injection Immunization with Different Doses

A total of 210 healthy largemouth bass were randomly divided into seven groups (30 fish per group). Fish in the immunization groups were IP injected with 50 µL of viral suspension at doses of 5 × 10^4^, 5 × 10^3^, 5 × 10^2^, 5 × 10^1^, 5 × 10^0^, or 5 × 10^−1^ TCID_50_/fish, while the control group was injected with 50 µL of PBS. At 21 dpi, all fish were challenged by IP injection of 50 µL SCRV-T6 at a dose of 5 × 10^5.0^ TCID_50_/fish. Mortality was monitored daily, and the relative percent survival (RPS) was calculated.

#### 2.4.2. Immersion Immunization with Different Doses

A total of 180 healthy largemouth bass were randomly assigned to six groups (30 fish per group). Fish in the immunization groups were immersed in viral suspensions at concentrations of 10^6^, 10^5^, 10^4^, 10^3^, or 10^2^ TCID_50_/mL for 1 h, followed by a 5 min rinse in clean water before being returned to the rearing tanks. The control group was subjected to the same procedure but immersed in clean water only. At 21 dpi, all fish were challenged by IP injection of 50 µL SCRV-T6 (5 × 10^5.0^ TCID_50_/fish). Daily mortality was recorded, and the RPS was determined.

#### 2.4.3. Transcriptional Analysis of Immune-Related Genes Post-Vaccination

Transcriptional profiles of immune-related genes (IL-10, IL-12, IFN-γ, TNF-α, and IgM) were analyzed by RT-qPCR. Spleen and kidney tissues were collected from five randomly selected fish per group at 0 h, 1, 2, 3, 7, 14, and 21 days post-vaccination (dpv). Total RNA was extracted using the Animal Total RNA Isolation Kit (FOREGENE, Chengdu, China) according to the manufacturer’s protocol. Total RNAs (5 μg) were reverse transcribed into cDNA using TransScripts II All-in-One First-Strand cDNA Synthesis (TransGen, Beijing, China). qPCR was performed using SuperMix for qPCR (ROX Plus) (TransGen, Beijing, China) on an ABI 7500 Real-time Detection System (Applied Biosystems, San Francisco, CA, USA). Each 20 μL reaction contained 2 μL cDNA, 0.4 μL of each primer (10 μM), and 10 μL of 2× Mix. The thermal cycling protocol consisted of initial denaturation at 95 °C for 1 min followed by 40 cycles of 95 °C for 15 s and 60 °C for 35 s. Gene expression levels were quantified using the comparative 2^−ΔΔCt^ method, where ΔΔCt= (Ct_, target gene_ − Ct_, reference gene_) vaccine − (Ct_, target gene_ − Ct_, reference gene_) control. 18S ribosomal DNA (18S rDNA) was used as the endogenous reference gene for normalization. All data were presented as means ± SD (n = 3). Primer sequences for all target genes are provided in Table 1.

#### 2.4.4. Serum Neutralization Assay

At 21 dpi, blood samples were collected from five fish in each group and allowed to clot at 4 °C for 1 h. Following centrifugation at 5000× *g* for 15 min, the supernatant serum was collected and heat-inactivated at 37 °C for 1 h. Serial two-fold dilutions of serum (ranging from 2^1^ to 2^10^) were prepared and mixed 1:1 (*v*/*v*) with viral suspension containing 10^2.0^ TCID_50_/mL. The serum–virus mixtures (100 µL/well) were added to 96-well plates containing confluent CPB cell monolayers. Plates were incubated at 28 °C in a non-CO_2_ incubator and monitored daily for CPE. Neutralizing antibody titers were calculated as the highest serum dilution that inhibited 50% CPE formation. Each plate included dedicated wells for positive controls (100 TCID_50_ virus) and negative controls (uninfected cells), with all conditions tested in triplicate.

### 2.5. Safety Test of the Attenuated Vaccine Candidate MSRV-0509

#### 2.5.1. Single Overdose Immunization

Healthy largemouth bass were randomly allocated into four groups (n = 30 per group) as follows: (1) IP injection group: Fish were injected with 50 μL of diluted viral suspension at a dose of 5 × 10^3.0^ TCID_50_/fish. (2) IP control group: Fish were injected with 50 μL PBS. (3) Immersion immunization group: Fish were exposed to viral suspension (10^7.0^ TCID_50_/mL) for 1 h, followed by 5 min rinsing in clean water before returning to rearing tanks. (4) Immersion control group: Fish underwent identical handling with clean water only. The triplicate experimental batch was performed for both injection and immersion. Mortality was recorded for 14 days.

#### 2.5.2. Reversion to Virulence Test

Largemouth bass (fish = 15) were injected intraperitoneally with MSRV-0509 (5 × 10^3.0^ TCID_50_/fish/0.05 mL). For the control group, fish were injected with 50 μL PBS. Ten fish were designated as the observation group and reared for 21 days at 28 °C, with five additional fish maintained as a sampling group. Daily mortality and clinical abnormalities were recorded in the observation group. At 24 h post-inoculation (hpi), liver, spleen, and kidney tissues were aseptically collected from the sampling group. MSRV detection and genomic copy numbers were quantified by TaqMan qPCR analysis. Parallel samples were homogenized in ice-cold PBS (1:10 *w*/*v*) and centrifuged at 5000 × rpm at 4 °C for 15 min. The supernatant was filtered through a 0.22 μm pore-size filter (Millex HV; Millipore, Burlington, MA, USA) to prepare challenge inocula. For serial passage, fifteen naive fish per group received intraperitoneal injections (0.1 mL) of inoculum derived from infected or control fish, following identical rearing protocols. This passage procedure was repeated for four cycles to establish MSRV-0509 infection in largemouth bass.

### 2.6. Statistical Analysis

The relative expression levels of immune-related genes across vaccinated groups were statistically analyzed using one-way ANOVA (GraphPad Prism 8.0, GraphPad Software, San Diego, CA, USA). If significant (*p* < 0.05), Fisher’s Least Significant Difference (LSD) post hoc test was subsequently applied to identify specific group differences.

## 3. Results

### 3.1. Determination of the Attenuated Rhabdovirus Strain

**Figure 1:**
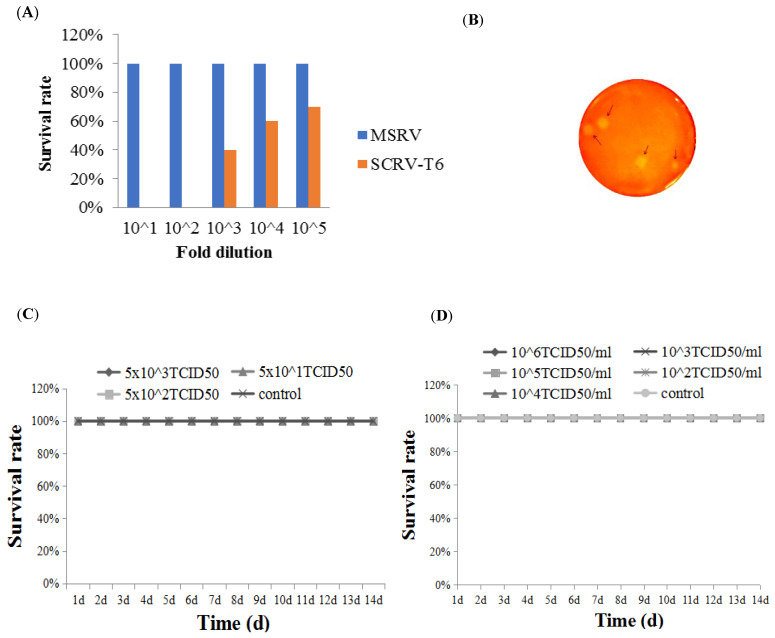
Determination of the attenuated MSRV strain. (**A**) Cumulative survival rates of largemouth bass by IP challenge with MSRV and SCRV-T6 at different dilutions. (**B**) Plaque purification. MSRV dilution in 10^−7^ formed plaques in CPB cell monolayers. Plaques were stained with neutral red solution for 3 h. Plaques are shown by arrowheads. (**C**) Survival rate of largemouth bass via IP injection or (**D**) immersion with different doses of MSRV-0509.

After five 10-fold serial dilutions (10^1^ to 10^5^) of MSRV and SCRV-T6, largemouth bass were intraperitoneally injected with 50 µL diluted virus per fish. The challenge tests showed cumulative mortality rates of 100%, 100%, 60%, 40%, and 30% for SCRV-T6 at doses of 5 × 10^6^, 5 × 10^5^, 5 × 10^4^, 5 × 10^3^, and 5 × 10^2^ TCID_50_/fish, respectively. However, no mortality was observed in any of the MSRV-challenged groups (a dose of 5 × 10^5.57^, 5 × 10^4.57^, 5 × 10^3.57^, 5 × 10^2.57^, or 5 × 10^1.57^ TCID_50_/0.05 mL per fish), suggesting that MSRV is an attenuated strain (Figure 1A). Subsequently, MSRV was purified by plaque purification. After five rounds of plaque purification, 10 clonal strains were harvested (Figure 1B) and the viral titer of MSRV-0509 was the highest (Table 2). Then the virulence of MSRV-0509 in largemouth bass was evaluated by both IP injection and immersion routes, and the results revealed that no mortality occurred in any tested group during the 14-day observation period, demonstrating MSRV-0509 to be an attenuated strain (Figure 1C,D).

### 3.2. Biological Characteristics of Clonal MSRV-0509 Strain

**Figure 2:**
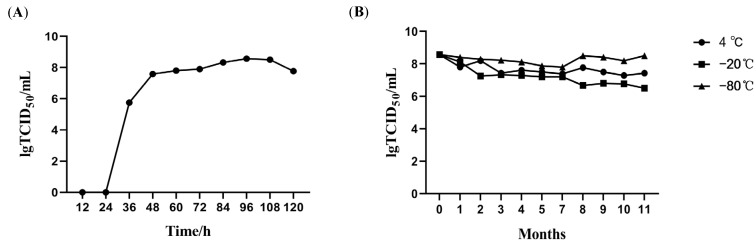
Biological characteristics of the clonal MSRV-0509 strain. (**A**) The growth curve of MSRV-0509 in CPB cells. (**B**) Titer changes of MSRV-0509 stored at different temperatures.

To establish the optimal inoculation dose, MSRV-0509 was inoculated at different multiplicities of infection (MOI of 1, 0.5, 0.1, 0.01, 0.001, and 0.0001), and viral titers were 10^6.410^, 10^7.000^, 10^7.289^, 10^7.800^, 10^8.000^, and 10^8.386^ TCID_50_/mL, respectively, indicating the highest viral titer was achieved at the lowest MOI (0.0001) (Table 3). Subsequently, MSRV-0509 replication in CPB was established. The viral growth curve exhibited four distinct phases: (1) a latent phase (0–24 h post-infection) with no significant titer increase, (2) an exponential growth phase (24–48 h) showing rapid viral replication, (3) a plateau phase (48–96 h) where viral titers peaked at 96 h (10^8.3^ TCID_50_/mL), and (4) a subsequent decline phase (>96 h) characterized by progressive titer reduction (Figure 2A). Then the thermal tolerance, chloroform sensitivity, acid/alkali resistance of MSRV-0509 were assessed. The results demonstrated that MSRV-0509 exhibits temperature-dependent reductions in viral infectivity, along with significant susceptibility to lipid solvents and acidic conditions (Table 4). Furthermore, MSRV-0509 stability after being stored at 4 °C, −20 °C, and −80 °C was assessed. The results demonstrated that storage at −80 °C provided optimal preservation, with viral titers remaining stable (≤0.5 log10 reduction) for over 11 months. In contrast, storage at 4 °C and −20 °C resulted in titer reductions of 1.142- and 2.071-log10, respectively, by month 11. These findings support −80 °C as the recommended storage condition (Figure 2B).

**Figure 3:**
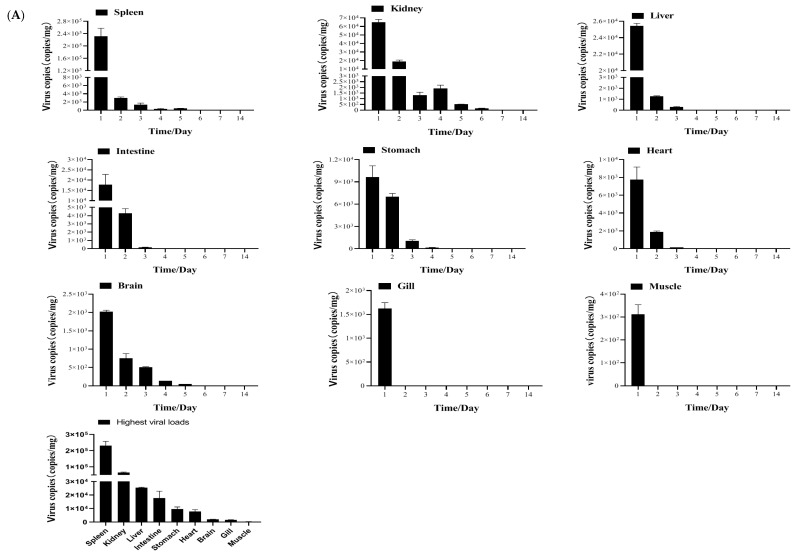

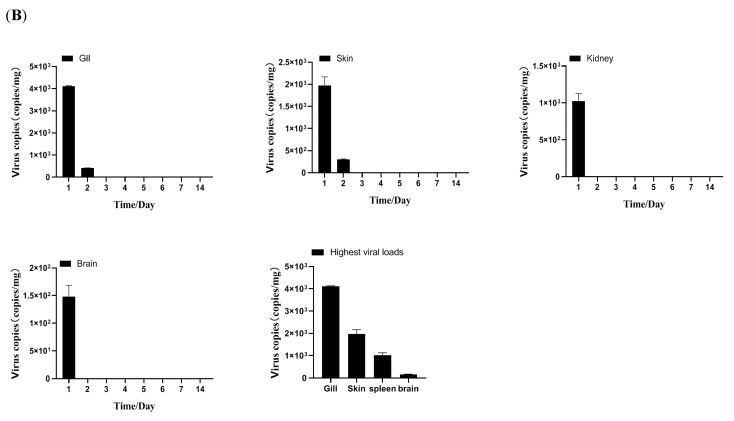
Tissue distribution and kinetics of MSRV-0509 in largemouth bass infected via (**A**) IP injection or (**B**) immersion. Virus copies in liver, spleen, kidney, heart, brain, stomach, intestine, gill, and muscle at different times were detected by qPCR.

Tissue distribution and kinetics of MSRV-0509 in largemouth bass via IP injection or immersion are shown in Figure 3A,B. After IP injection challenge, MSRV-0509 exhibited distinct tissue distribution and kinetics in largemouth bass. Viral loads peaked in nine tissues at 1 dpi, with 2.31 × 10^5^ copies/mg in spleen tissue, of 6.50 × 10^4^ copies/mg in head kidney tissue, 2.55 × 10^4^ copies/mg in liver tissue, 1.78 × 10^4^ copies/mg in intestine tissue, 9.67 × 10^3^ copies/mg in stomach tissue, 7.76 × 10^3^ copies/mg in heart tissue, 2.02 × 10^3^ copies/mg in brain tissue, 1.62 × 10^3^ copies/mg in gills, and 3.12 × 10^2^ copies/mg in muscle. All tissues showed progressive viral load reduction after 2 dpi, becoming undetectable by 6 dpi. Comparative analysis of peak viral loads revealed the spleen as the primary target organ. But, after immersion infection, MSRV-0509 detection was limited to four tissues of gills, skin, kidney, and brain, with no detectable viral load in heart, liver, spleen, intestine, stomach, and muscle tissues. Viral loads also peaked at 1 dpi in all positive tissues, with 4.12 × 10^3^ copies/mg in gills, 1.97 × 10^3^ copies/mg in skin, 1.13 × 10^3^ copies/mg in the kidney, and 1.47 × 10^2^ copies/mg in the brain. Notably, viral clearance occurred rapidly, with all tissues becoming negative by 3 dpi.

### 3.3. Evaluation of Protective Efficacy of Attenuated Vaccine Candidate MSRV-0509

**Figure 4:**
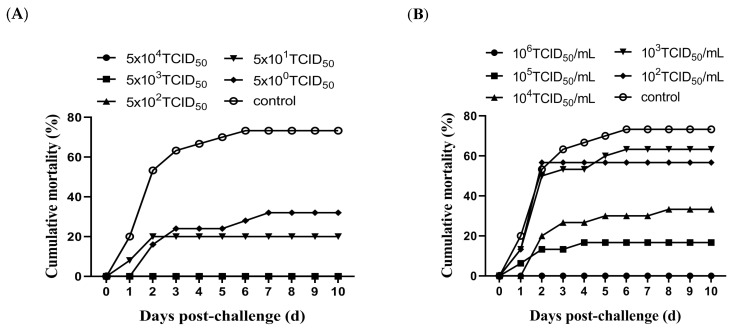
Protective efficacy of attenuated MSRV-0509 vaccine against virulent SCRV challenge in largemouth bass. Cumulative mortality of largemouth bass post-challenge with virulent SCRV after (**A**) IP injection or (**B**) immersion vaccination at different doses.

To establish the effective immunization dose of the attenuated MSRV-0509 strain, largemouth bass were administered the vaccine via IP injection or immersion at different doses. As shown in Figure 4A, largemouth bass intraperitoneally administered with doses of 5 × 10^4^, 5 × 10^3^, and 5 × 10^2^ TCID_50_/fish obtained complete protection (RPS = 100%). But the RPS values of lower-dose groups (5 × 10^1^ and 5 TCID_50_/fish) were 79.2% and 66.7%, respectively. For immersion groups, largemouth bass exhibited RPS values of 100%, 75.0%, 52.3%, 9.5%, and 14.3% for vaccine doses of 10^6^, 10^5^, 10^4^, 10^3^, and 10^2^ TCID_50_/mL, respectively (Figure 4B). These results demonstrated that the attenuated MSRV-0509 strain conferred significant immune protection in vaccinated fish, and the minimum immunizing dose was 5 × 10^2^ TCID_50_/fish for IP injection and 10^6^ TCID_50_/mL for immersion.

### 3.4. Transcription of Immune-Related Genes and Serum Neutralization in Largemouth Bass Post-Vaccination

**Figure 5:**
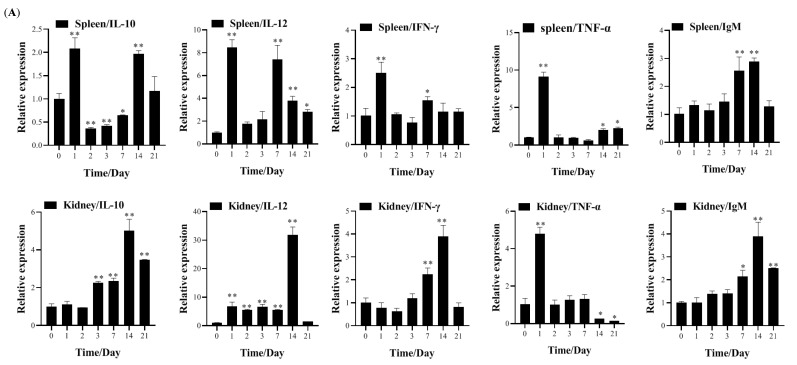

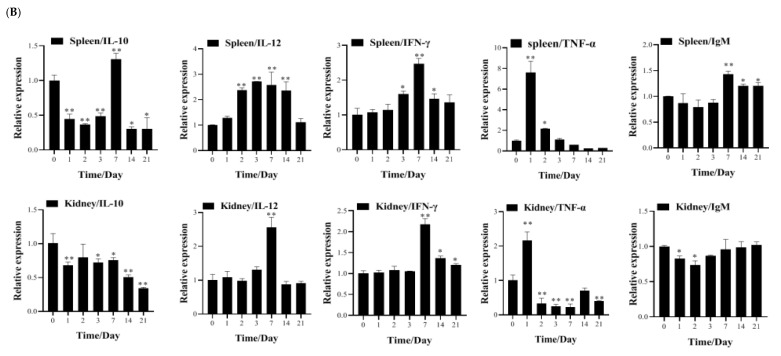
Relative expression of immune factors in spleen and kidney of largemouth bass vaccinated with MSRV-0509 via (**A**) IP injection or (**B**) immersion vaccination. Total RNA was isolated from spleen and kidney tissues collected from vaccinated and control groups at 0, 1, 2, 3, 7, 14, and 21 dpv. Transcript levels of immune-related genes (IL-10, IL-12, IFN-γ, TNF-α, and IgM) were quantified by qRT-PCR. Statistical significance is indicated as follows: * *p* < 0.05, ** *p* < 0.01. Data are presented as means ± SE (N = 3).

To investigate the immune response mechanisms induced by the live-attenuated MSRV-0509 in largemouth bass, the relative expression levels of Th1 cell-mediated immune-related genes (IL-12 and IFN-γ), Th2 cell-mediated immune-related genes (IL-10 and IgM), and inflammatory factors (TNF-α) were detected. For IP injection groups, expression of IL-10, IL-12, IFN-γ, and IgM in the spleen and kidney showed an up-regulation trend post-immunization. But TNF-α in two tissues peaked sharply at 1 dpv, followed by gradual decline (Figure 5A). But for immersion groups, IL-10 expression in the spleen and kidney showed a declining trend post-immunization. IL-12 and IFN-γ expression in the spleen and kidney showed a gradual increase and peaked at 3 or 7 dpv, followed by gradual decline. IgM expression showed a modest elevation in the spleen or remained stable in the kidney throughout the 21-day observation period. TNF-α expression, similar to that of the IP group, peaked sharply at 1 dpv, followed by gradual decline (Figure 5B). The serum neutralization test results showed that at a serum dilution of 1:64 for the IP group or 1:8 for the immersion group, while no CPE was observed in negative or positive controls, indicating a serum antibody titer of 1:64 for IP injection and 1:8 for immersion (Table 5). These results demonstrated that MSRV-0509 via IP injection activated Th1-type cellular immunity and Th2 humoral immunity, while immersion vaccination preferentially activated Th1-type cellular immunity.

### 3.5. The Safety Evaluation of Single Overdose Vaccination and Virulence Reversion

For single overdose vaccination, largemouth bass were either immersed in the MSRV-0509 strain (10^7^ TCID_50_/mL) or intraperitoneally injected with 5 × 10^3^ TCID_50_/fish (three replicates per group). After 14 days of continuous observation, no mortality was recorded (Table 6), confirming the safety of the MSRV-0509 strain even at overdose vaccination levels. For virulence reversion testing, liver, spleen, and kidney homogenates were prepared based on the tissue distribution results. Five serial passages in largemouth bass revealed no clinical abnormalities or mortality following MSRV-0509 infection during the observation period. Viral detection by qPCR was limited to the first and second passages and no virus was detected in subsequent passages (Table 7). These results demonstrated no evidence of virulence reversion in the attenuated MSRV-0509 candidate vaccine after fish-to-fish serial passages.

## 4. Discussion

The development of effective vaccination strategies is crucial for sustainable aquaculture, particularly for economically important species like largemouth bass that are vulnerable to viral diseases. In this study, we successfully generated and characterized an attenuated MSRV strain (MSRV-0509) with potential as a live-attenuated vaccine candidate. Our findings demonstrate that MSRV-0509 exhibits several key characteristics of an effective vaccine strain, including reduced virulence, stable biological properties, and the ability to induce protective immunity without evidence of virulence reversion.

In our current study, MSRV-0509 was completely inactivated by heating at 56 °C for 30 min, or chloroform exposure for 10 min, while its activity was reduced by pH3.0 treatment for 120 min. Gui et al. investigated hirame rhabdovirus (HIRRV) and found that treatment at 56 °C for 60 min, pH2.0 for 60 min, or chloroform completely inactivated the virus [16]. Two fish rhabdoviruses exhibited similar physicochemical characteristics. The viral growth kinetics of MSRV-0509 in CPB cells followed a typical replication curve, with peak titers reaching 10^8.3^ TCID_50_/mL, suggesting viral production potential for vaccine manufacturing [17]. The temperature stability tests demonstrated that viral titers remained stable (≤0.5 log10 reduction) when stored at −80 °C for 11 months, while storage at 4 °C and −20 °C resulted in a 1.142 and 2.071 log10 titer reduction over the same period. These findings suggest the need for lyophilization protective agents to enhance stability under refrigerated conditions [18]. Tissue distribution studies revealed interesting route-dependent infection patterns. IP injection administration resulted in broader systemic distribution with the highest viral loads in the spleen, while immersion exposure led to localized infection primarily in the gills and skin, providing important implications for vaccination strategies. Moreover, the rapid clearance of MSRV-0509 (undetectable by 3–6 dpi) was found, suggesting the limited persistence in largemouth bass.

Until now, several MSRV vaccines for largemouth bass vaccination have been investigated. Guo et al. developed a single-walled carbon nanotube (SWCNT)-conjugated G-protein vaccine that induced 70.1% RPS via intramuscular (IM) immunization [19]. Similarly, a bacterial nanocellulose (BNC)-displayed G-protein vaccine achieved 66.7% RPS following IM administration [20]. For oral vaccination, a yeast surface-expressed G-protein subunit vaccine demonstrated equivalent efficacy (66.7% RPS) [21]. In contrast, intramuscular delivery of pcDNA3.1-G DNA vaccine yielded lower protection (48.5% RPS) [21]. Subsequent studies showed that pcDNA3.1-G2, containing a partial G-gene sequence (382–795 bp), could elicit significantly higher protection (82.5% RPS) [22]. In this study, the immunization experiments demonstrated that MSRV-0509 could provide complete protection (100% RPS) by both immersion (10^6.0^ TCID_50_/mL) and intraperitoneal (5 × 10^2.0^ TCID_50_/fish) routes. The immersion vaccination may be more suitable for mass vaccination of juvenile fish, while injection could be reserved for broodstock.

Both humoral and cellular immune responses play essential roles in viral defense and clearance. The humoral immune response, mediated by Th2 cells, produces antibodies, while cellular immunity is primarily driven by Th1 cells [23]. IL-10 is predominantly secreted by Th2 cells [24] and IL-12, a critical regulator of Th1 responses that enhances IFN-γ production [25]. In this study, expression of IL-10, IL-12, IFN-γ and IgM revealed that MSRV-0509 administration elicited differential immune responses depending on the vaccination route. Immersion vaccination preferentially stimulated Th1-type cellular immunity, whereas intraperitoneal injection activated both Th1 and Th2 responses. Future studies should elucidate the precise immunological mechanisms involved.

The vaccine safety is the fundamental consideration in live-attenuated vaccine development, especially virulence reversion. Evaluations of virulence reversion through serial back-passage studies in vivo have confirmed this for the modified live Flavobacterium columnare vaccine [13] and the attenuated Cyprinid herpesvirus 2 (CyHV-2) vaccine [5], with no observed reversion to virulence. Our serial passage experiments also found no evidence of virulence reversion, demonstrating that MSRV-0509 exhibited no virulence reversion. Furthermore, overdose vaccination studies validated MSRV-0509’s safety as a promising live-attenuated vaccine candidate. We speculated that MSRV-0509 induced only limited TNF-α production, indicating that it does not elicit excessive inflammatory responses, and the restricted tissue tropism and rapid clearance contribute to its excellent safety.

In summary, MSRV-0509, a promising attenuated vaccine candidate, was screened. We found that it has good immunogenicity and safety for largemouth bass against virulent rhabdovirus via immersion vaccination and intraperitoneal injection. Therefore, MSRV-0509 is a potential live vaccine candidate against MSRV disease.

## Figures and Tables

**Table 1:** Primers used for qRT-PCR detection of gene expression.

Primer Name	Sequence (5′-3′)
TNF-α-F	ACTTCGTCTACAGCCAGGCA
TNF-α-R	AGTAACGCGAGACCCTGTGG
IFN-γ-F	CGCCTCCATCAGCACCGACA
IFN-γ-R	CGGCAGCTCCCACAATGCTT
IL-10-F	ACAACCAGTGCTGCCGTT
IL-10-R	GCAGCGCTGTGTCTAAGTCA
IL-12-F	TCTTCCATCCTTGTGGTCTTCC
IL-12-R	CAGTTCCAGGTCAAAGTGGTC
IgM-F	ATTGTCAGGTCCATCGGGC
IgM-R	TACCGAATCACCTCGAGAGGGA
18S-F	CATTCGTATTGTGCCGCTAGA
18S-R	CAAATGCTTTCGCTTTGGTC

**Table 2:** The viral titers of 10 clonal MSRV strains.

Numbers	1	2	3	4	5	6	7	8	9	10
Titer (TCID_50_/mL)	10^8.52^	10^8.50^	10^8.39^	10^8.50^	10^8.59^	10^8.57^	10^8.36^	10^8.20^	10^8.20^	10^8.00^

**Table 3:** Virus titers at different MOIs.

MOI	1.0	0.5	0.1	0.01	0.001	0.0001
lgTCID_50_/mL	6.410	7.000	7.289	7.800	8.000	8.386

**Table 4:** MSRV-0509 titers after different treatments.

Treatment Conditions		lgTCID_50_/mL
Temperature (°C)	28	8.667
	34	8.410
	40	8.289
	46	7.750
	52	4.000
	56	3.574
	60	0
Chloroform	Chloroform	0
	Control	8.0
pH	3.0	3.563
	5.0	7.800
	9.0	8.289
	Control	8.500

**Table 5:** CPE observation following serum neutralization.

Group	Serum Dilution Ratio										Negative Control	Positive Control
	1:2	1:4	1:8	1:16	1:32	1:64	1:128	1:256	1:512	1:1024		
IP injection Immersion	−	−	−	−	−	−	+	+	+	+	−	+
	−	−	−	+	+	+	+	+	+	+	−	+

Note: “+” represents CPE, “−” represents no CPE.

**Table 6:** Mortality of largemouth bass vaccinated at overdosed via IP injection or immersion.

Group	Vaccination Route	Dose (TCID_50_)	Fish Number/Group	Number of Deaths			Mortality (%)		
				First Batch	Second Batch	Third Batch	First Batch	Second Batch	Third Batch
Vaccine	IP injection	5 × 10^3^/0.1 mL/fish	30	0	0	0	0	0	0
Control		PBS/0.1 mL/fish	30	0	0	0	0	0	0
Vaccine	Immersion	10^7^, for 1 h	30	0	0	0	0	0	0
Control		PBS, for 1 h	30	0	0	0	0	0	0

**Table 7:** Viral load detection in virulence reversion test.

Passage	Ct Value	Viral Load (Copies/mg)
1	33.841	511.8259
2	34.007	459.273
3	ND	0
4	ND	0
5	ND	0

“ND” meaned not detected.

## Data Availability

The data that support the findings of this study are available from the corresponding author upon reasonable request.
